# Sociodemographic Characteristics and Stress of People from Spain Confined by COVID-19

**DOI:** 10.3390/ejihpe10040077

**Published:** 2020-12-03

**Authors:** Susana Rodríguez, Antonio Valle, Isabel Piñeiro, Carolina Rodríguez-Llorente, Estefanía Guerrero, Ludmila Martins

**Affiliations:** Department of Psychology, Universidade da Coruña, 15071 A Coruña, Spain; susana.rodriguez1@udc.es (S.R.); antonio.valle@udc.es (A.V.); isabel.pineiro.aguin@udc.es (I.P.); estefania.guerrero@udc.es (E.G.); l.martins@udc.es (L.M.)

**Keywords:** COVID-19, stress, control of stress, confinement

## Abstract

This study responds to the need to explore the individual characteristics that may help us to understand the levels of stress involved in the significant COVID-19-related restrictions to people’s daily lives. In order to understand levels of stress and stress control during the COVID-19 confinement, 1269 people from Spain (17.5% men) aged between 18 and 70 completed the Perceived Stress Scale (PSS-14). The results indicated that people aged under 40, and especially those under 25, women, and those on low incomes reported higher rates of confinement stress. The nature of where people live, and their working situation during confinement also contributed to people’s stress response, although with lower levels of impact. In this context, our study suggests that the levels of stress in those who combine remote working with in situ working were lower than those who had other working conditions. Our study contributes significant information to understanding the effects of confinement, and its results may be used to inform intervention tools and programs.

## 1. Introduction

Unprecedented restrictions have been put in place on people’s daily lives all over the world to contain the COVID-19 pandemic which may be triggering psychological symptoms of anxiety, stress, and depression [1]. Confinement (lockdown or quarantine) at home, the containment measure for the virus chosen by most governments, may have had immediate and long-term repercussions on people’s mental health and quality of life [2,3], and has been positively associated with depressive symptoms [4,5], anxiety, and stress in situations of crisis [6].

Stress is known to be a common reaction to catastrophic or emergency situations [7], and despite the effects of isolation due to epidemics being confirmed in certain geographical areas [2,8,9], information about the stress response and control of stress during mass confinement caused by a pandemic like the one we are experiencing now is limited [10]. Quarantine may be an unpleasant experience because of separation from loved ones, loss of liberty, uncertainty about illness, and boredom [11]. This has led to the potential psychological impacts of confinement during the COVID-19 pandemic [12,13,14] being an object of interest in most countries. In view of this situation, this study aimed to evaluate the impact of confinement on people’s quality of life, considering the differences that may be due to sociodemographic conditions such as sex, age, where people live, and income levels [13]. It is important to establish profiles of those who have suffered higher levels of stress and those who have managed it worse, given the potential physical and mental impact in the short and long term [15,16,17].

### 1.1. Stress and Confinement

In psychology, the transactional model of stress and coping from Lazarus [18] considers stress as the behavioral result of the relationship between the coping resources a person has available and the demands of the situation they are in. Lazarus [18] highlights the importance of individual differences in the experience of stress, understanding that the stress relationship is constantly changing as the result of continual interaction between the person and their surroundings. In previous epidemics, various studies reported elevated levels of stress and anxiety in the population [19,20], leading to behaviors that were not adaptive as well as socially unsuitable [12,21,22].

In fact, generalized outbreaks of similar infectious diseases have been associated with psychological distress and symptoms of mental illness [8]. During the Korean MERS-CoV outbreak in 2015, patients in isolation [9] and healthcare professionals in public hospitals [2] experienced high levels of stress. Similarly, during the Ebola outbreaks in Sierra Leone in 2014 and the Democratic Republic of the Congo in 2018, medical personnel reported high levels of anxiety [23].

In the current COVID-19 crisis, moderate and severe anxiety as well as symptoms indicating the psychological impact of confinement have been recorded in the population [24,25]. More than a quarter of the Chinese population reported moderate to severe levels of stress [1,26], and in Iran, studies have highlighted the role of unpredictability, uncertainty, the seriousness of the illness, disinformation, and social isolation on the experience of stress and mental ill-health [3]. In Japan [2], negative effects have been confirmed on wellbeing, along with high levels of fear and panic in the population in general, with greater risk for infected patients and their families, individuals with physical or psychiatric illnesses, and healthcare workers.

### 1.2. Stress and Sociodemographic Factors 

A series of sociodemographic factors such as gender, age, educational level, and working situation seem to be differentially linked to levels of perceived stress during the COVID-19 crisis [4,6,10,27] suggesting that cultural differences may need specific attention in this context [11]. Studies performed in the context of this pandemic, for example, report that vulnerable groups include older adults [28], with that vulnerability being more marked in those suffering psychiatric issues, the homeless [29], migrant workers [30], pregnant women [31], and Chinese students who were abroad [32]. In addition, patients with preexisting mental disorders may have a higher risk of relapse or new episodes due to the stress associated with COVID-19 outbreaks [33].

On similar lines, studies seem to suggest that being a woman, having a low socioeconomic level, having interpersonal conflicts, frequently using communication media, and perceiving low levels of social support could increase the risk of developing psychological problems as a result of confinement situations [34]. Regarding gender differences, women seem to suffer more anxiety. Although it appears that men show lower stress levels than women [35,36], which might be related to a better resilience in them [37], the literature has not reached clear agreement on the matter [38,39]. 

In terms of age, a study in 41 countries on the perception of stress caused by COVID-19 suggests that older people report the lowest levels of anxiety [40], and that being under 45 is a risk factor for perceived stress. It is possible that the highest levels of stress, depression, and anxiety are found in young people aged between 18 and 25, mostly students [41], and that, in general, the younger population exhibits higher levels of anxiety and a greater prevalence of stress [42]. Notwithstanding this, we have also found data indicating levels of stress to be equally high for younger people and adults [39]. Moreover, older people suffer higher levels of stress in this particular context [43], which increases with age, reaching the highest levels for 55–65 year-olds [44]. For this reason, addressing age in terms of the confinement stress response and control continues to be important.

With respect to civil status, our study will attempt to confirm, as the ad hoc literature maintains, that married people or those living with partners tend to experience lower levels of stress due to confinement than those who are single [36] or not living with partners [45]. We also hypothesize that the level of education in a population may be related to the stress response and/or control of stress due to confinement [23]. People with higher levels of education would exhibit lower levels of anxiety and stress [45] and better control of the stress of confinement than those with lower levels of education.

Another variable we examine in this study is the type of residence during the confinement, as we expect people who live in bigger houses, with more space, to exhibit lower levels of stress and anxiety during confinement due to COVID-19. It has also been found that people who have open air space in their properties exhibited slightly lower levels of psychological impact in this period compared to those who had not outside space [45].

Lastly, in terms of the diverse, significant impact of the COVID-19 confinement on the working lives of affected populations, we expect to see an interaction between people’s work situations and income levels and their reported levels of stress [24,25]. One of the groups at highest risk of suffering more stress due to the confinement and generally managing the situation worse would be those who have had their work and ability to generate income affected [42,46].

Based on the above, the objective of our study is to identify the differences in perceived stress and control of stress as a function of the sociodemographic characteristics of the population confined by COVID-19 (age, gender, civil status, education, place of residence, income and work situation during confinement). In particular, given the literature reviewed, we expect to find differences in the perception and control of stress with respect to gender, age, civil status, educational level, income level, type of residence, and work situation during the confinement. Furthermore, in this study, the validity and reliability of the PSS-14, the instrument used for data collection, is also explored.

## 2. Materials and Methods

### 2.1. Participants

During confinement, 1269 people (17.5% men) voluntarily and anonymously completed an online questionnaire designed to capture their response to situational stress and the coping strategies they were using. Participants were aged between 18 and 70 years old (M_age_ = 38.76; SD = 10.58). The Spanish government declared a highly restrictive shielding of the population from the 15th of March until mid-May, when lock-down became more flexible. The population of Spain was allowed to leave their house for food sourcing exclusively. Only basic needs services employees attended their work places while others teleworked. As a result of the confinement restrictions, many people went on temporary suspension of employment.

At the time of the confinement, most of the participants were living with their spouse and children (47%), 14.5% were living alone, and almost 20% were in a couple but without children. Nearly 6% of participants were confined with their parents. More than half of the respondents were married or in a stable partnership, 25.7% were single, and almost 7% were divorced or separated. A small minority (1.3%) were widowed. 

More than half of the sample in this study were full-time workers at the time of confinement and 17.1% were working part-time/temporarily. On the other hand, 8.3% were not working prior to the lock-down, and almost 4% were studying/preparing public exams when the confinement was decreed. Most respondents (66.7%) reported spending the confinement in urban surroundings while the rest were in rural (21.8%) or residential/suburban (11.5%) areas. Just less than half (48.5%) of the participants worked remotely during the confinement, 15.7% regularly attended their places of work, and a similar percentage were temporarily suspended from work (21.9%). Only 6.8% of the sample reported having lost their jobs, and 7.1% combined remote working and attending their place of work at some point.

Most participants reported being within six or seven weeks of confinement (49%), 34% had been confined for four or five weeks when they responded to the questionnaire, and almost 15% informed being confined for more than seven weeks. 

### 2.2. Instruments

We examined a series of sociodemographic factors to examine their relationship to the stress response to confinement. In order to evaluate the levels of stress, we used the 14-item Perceived Stress Scale (PSS-14) created by Cohen, Kamarck and Mermelstein [47]. This is a 5-point Likert-type scale, ranging from 0 to 4 (where 0 = never and 4 = very often), that has traditionally been reported to show good internal and structural consistency [48,49,50,51].

In agreement with the theory and psychometric studies with the PSS (both PSS-14 and PSS-10), in this study, it demonstrated a two-factor structure made up of positively and negatively worded elements [52] which, with eigenvalues over 1, explain 52.99% of the variance. Factor analysis for the whole sample allowed us to differentiate between Control of Stress during the confinement period (α = 0.81) (Example items: *During the period of confinement… How often have you felt that you have successfully dealt with significant changes in your life? How often have you felt confident in your ability to manage your personal problems?* and *How often have you successfully dealt with daily preoccupations?*) and Perceived Stress (α = 0.85) during confinement (Example items: *During the period of confinement… How often have you felt anxious? How often have you felt overwhelmed by something that has happened unexpectedly?* and *How often have you felt incapable of controlling the important things in your life?*). Both chi-square from the transformation of the determinant of the correlation matrix (Bartlett’s sphericity of 0.000) and the size of the correlation coefficients (KMO = 0.921) indicated the suitability of the factorial structure.

### 2.3. Procedure

On the 18th April 2020, we published a direct link to the questionnaire on various social networks and via various media, both print and digital, to call for participation in the study. We followed the recommendations of the Committee of Ethics in Research and Teaching from the University of A Coruña and the Declaration of Helsinki, complying with each aspect related to voluntary, anonymous participation and data protection.

The evaluation tools were presented as an online survey which took approximately 15 min to complete. We created the data collection instrument using the Microsoft Forms platform, which included informed consent, a sociodemographic questionnaire, and amongst other questions, the PSS-14 to measure perceived stress and stress control.

We obtained the sample using non-probabilistic exponential snowball sampling. The link to the survey was sent via email, social networks (WhatsApp, Facebook, and Instagram), and online written media. The sole inclusion criterion was that participants had reached the age of majority in each applicable country, there were no other exclusion criteria applied.

A month after the questionnaire link publication, on the 19th of May 2020, access to the survey was closed.

### 2.4. Data Analysis

Apart from examining the descriptive statistics for the items and the relevant factorial analysis (validity and reliability) of the PSS-14, we also performed an analysis of variance (ANOVA) to explore the differences in the levels of perceived stress during the confinement as a function of a mix of sociodemographic variables. We also examined the differences in levels of stress control with respect to the same set of sociodemographic variables. We used the criteria established by Cohen [53] to interpret effect sizes. According to that, an effect is small when η_p_^2^ = 0.01 (*d* = 0.20), moderate when η_p_^2^ = 0.059 (*d* = 0.50), and the effect is large when η_p_^2^ = 0.138 (*d* = 0.80). All the data created in this research is available at Zenodo [54]. 

## 3. Results

### 3.1. Differences in Stress According to Gender, Age, and Civil Status

We found significant differences in perceived stress between men and women confined at home due to COVID-19 (*F*_(1,900)_ = 64.960; *p* < 0.001; η_p2_ = 0.049), but not in the reported levels of control of stress. Women reported feeling more anxious and overwhelmed than men during the confinement, but dealt with changes and managed confinement similarly to men.

**Figure 1:**
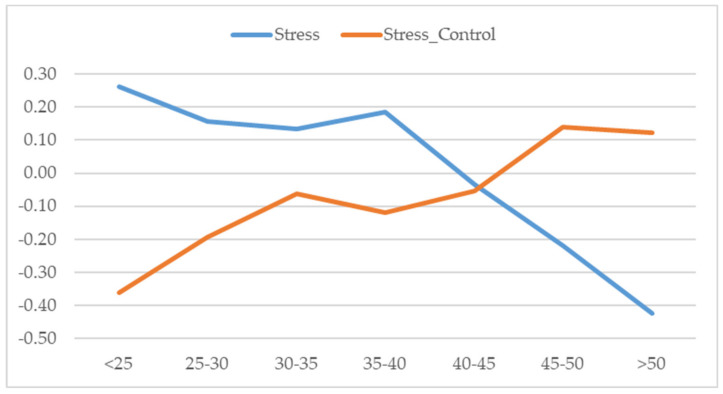
Differences in perceived stress and control of stress by age.

The results also suggested significant differences both in the stress response (*F*_(6,898)_ = 12.152; *p* < 0.001; η_p2_ = 0.055) and the level of control of stress (*F*_(6,920)_ = 4.965; *p* < 0.001; η_p2_ = 0.023) as a function of age (see Figure 1).

Note: Significant differences 0.05 (Scheffé): Stress: (<25)-(45–50); (<25)-(>50); (25–30)-(45–50); (25–30)-(>50); (30–35)-(>50); (35–40)-(45–50); (35–40)-(>50); (40–45)-(>50); Stress Control: (<25)-(45–50); (<25)-(>50)

Post hoc (Scheffé) analysis showed that perceived stress was significantly lower in those aged over 40 than those under 40, and the older group managed confinement better. The highest levels of self-reported stress and the worst control of stress was in the under-25s.

However, no significant differences have been found either in stress perception or in stress control according to civil status.

### 3.2. Differences in Stress by Education Level, Residence, Income, and Work Situation During Confinement

Although the effect sizes were small, we did find significant differences between people in control of stress with respect to their education (*F*_(3,401)_ = 4.990; *p* < 0.01; η_p2_ = 0.012). Participants reporting university level qualifications had higher mean scores in the PSS-14 positive control of stress factor, whereas those with no qualifications had the lowest scores.

Although differences in control of stress were not significant, the perception of stress during confinement was different (*F*_(2,748)_ = 5.558; *p* < 0.01; η_p2_ = 0.009) depending on where people lived. The results indicated that those who were confined in rural and urban environments reported higher levels of stress than those in residential/suburban surroundings.

Noting the positive correlation between income level and age (r = 0.35), we also found significant differences in perceived stress (*F*_(5,730)_ = 10.459; *p* < 0.001; η_p2_ = 0.040) and in control of stress (*F*_(5,391)_ = 9.901; *p* < 0.001; η_p2_ = 0.038) in confinement as a function of income level.

**Figure 2:**
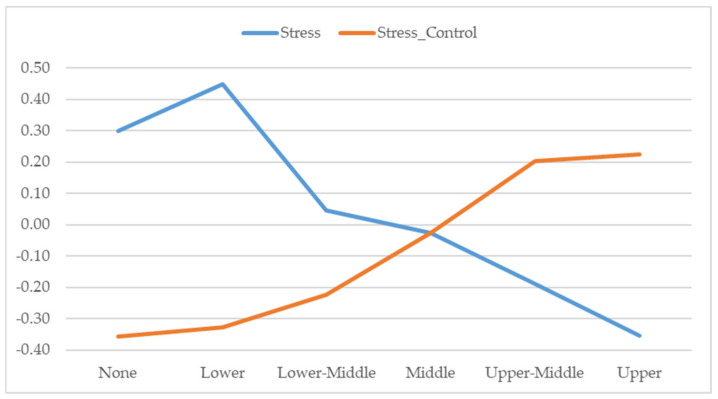
Differences in perceived stress and control of stress by income level.

As Figure 2 shows, perceived stress was greater in those with no or low incomes, and reported stress levels were lower the higher the reported income. Similarly, post hoc analysis showed that management of stress during confinement was worse for those with lower incomes and was reportedly easier the higher the economic level (see Figure 2).

Note: Significant differences 0.05 (Scheffé): Stress: None-Upper-Middle; Lower-Middle; Lower-Upper-Middle. Stress Control: None-Upper-Middle; Lower-Upper-Middle; Lower-Middle-Upper-Middle. 

Lastly, the working situation during confinement was also related to the stress response (*F*_(4,918)_ = 4.914; *p* < 0.01; η_p2_ = 0.020) and the control of stress during that period (*F*_(4,928)_ = 4.017; *p* < 0.01; η_p2_ = 0.016).

**Figure 3:**
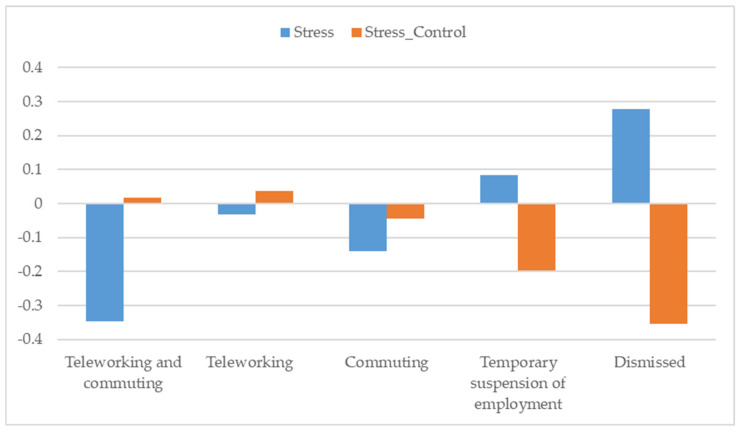
Differences in perceived stress and control of stress by working situation during confinement.

As Figure 3 shows, the lowest stress response was reported by those who combined remote working with attending the workplace during confinement. As expected, those who had lost their jobs during the confinement exhibited the highest stress response and the worst control of stress during this time (See Figure 3).

Note: Significant differences 0.05 (Scheffé): Stress: Teleworking and commuting-Temporary suspension of employment; Teleworking and commuting-Dismissed. Stress Control: Teleworking-Dismissed. 

## 4. Discussion

The confinement, which was a preventive measure in the face of the COVID-19 pandemic, is a sufficiently novel context in which to attempt to explore the individual characteristics that can help us to understand the level of stress involved in people being confined in their homes.

Our results confirmed that age, gender, and income level are the sociodemographic factors with the greatest explanatory potential for perceived stress in people confined due to COVID-19 [55]. Those aged under 40 [27,39,40] and particularly those under 25 [41], women [31,35,36,43,56], and those on low incomes [24,42,45,46] reported higher rates of stress in confinement. In terms of age, our results indicated that younger people tended to have more difficulties controlling the stress of confinement, and demonstrated greater difficulties dealing with the changes and adapting to them [56]. At the same time, having a low income level not only contributed to stress in confinement, but also to its management. Difficulties in obtaining basic supplies and protective equipment, and in accessing information and resources, may increase stress levels in isolation and, therefore, will possibly worsen how stress is managed during confinement [11].

It is important to note that despite the differences in the reported stress response between men and women, we did not find differences in terms of gender with regard to the control of stress in confinement [10]. For women, the current situation of teleworking [38]—with higher risks of losing their jobs than men [43]—when it is women who often have to take on care activities, raising children, and housework [43,57], may contribute to higher perceived levels of stress. Despite this evidence, our results did not suggest, a priori, gender differences in the ability to control stress during confinement.

Although effect sizes were smaller, where people live and their working situation during confinement also contributed to the stress response, confirming previous results [55].

Compared to people who live in rural or urban environments, those who spent confinement in residential/suburban environments had lower levels of stress. It is possible that those who live in bigger houses with more space generally exhibited lower levels of stress and anxiety [45]. It is worth noting that in our study, those who spent confinement in rural surroundings reported similar rates of stress to those who were in urban surroundings. Perhaps in the rural environment, the lack of connection with public services, worse healthcare provision against the pandemic [29], and a lack of means to keep in contact with loved ones [11] increase the perception of vulnerability and isolation, affecting the perception of stress.

The economy has also been severely affected by the COVID-19 crisis, causing uncertainty and meaning many people being afraid that they may lose their jobs [45]. In this unusual situation, both income and work conditions may be risk factors for stress [56,58]. Our results suggested that levels of stress in those who were able to combine teleworking with attending the workplace were lower than for those with other working conditions. This combination was also associated with better control of stress during confinement. Our results indicated that there was no significant difference in stress between those who worked from home during confinement and those who regularly attended their place of work. Finally, as we expected, the highest levels of stress and the worst control of stress was in those who had lost their jobs due to the pandemic.

Our hypothesis about the relationship between civil status and stress has not been supported, to the extent that single people reported similar levels of stress than those with a different civil status [36,45]. Being confined with a partner, having someone with whom to share the experience and have frequent contact with may not be associated with better control of stress [44]. According to our results, people who were separated or divorced demonstrated similar levels of control of stress during confinement to those who were married, living with a partner, or single.

Finally, although the effect sizes were small, our results showed that, although we did not find differences in levels of stress, those with higher qualifications were better able to manage stressful situations, such as those involved in confinement, than people with low educational qualifications [59].

The results of our study are a contribution to understanding the perception and control of stress during the confinement in the current pandemic. Understanding the relationships between sociodemographic factors and stress will allow us to establish plans of action, intervention, and application of measures to help people overcome the effects of the crisis. Similarly, the data may be important for forming specialist teams and may contribute to the design of tools for professionals and volunteers to work in these contexts.

The study does have some limitations that must be borne in mind when interpreting the results and for future research. Firstly, it was a quantitative, transversal study, and it did not allow longitudinal follow-up of the stress process during the different phases of confinement. The questionnaire was administered online, which meant it excluded a good proportion of the population who cannot access the internet or do not have the relevant electronic devices. In addition, the use of a non-probability convenience sample may not offer a good representation of the population of Spain. Hence, the results of this study must be taken into consideration carefully.

## 5. Conclusions

This study showed that during the confinement, people experienced differing levels of stress and managed it with varying effectiveness depending on their demographic characteristics:

- Although women reported higher levels of stress, probably due to the traditional care roles that they played in the family, they managed that stress in a similar way to men.
- In those who were over 40 years old, levels of stress were lower, and control of stress was better.
- People living in urban and rural areas reported high levels of stress, but those living in semi-urban/suburban or residential areas (with open spaces and accessible resources) reported lower levels.
- The higher the level of income, the lower the stress and the better the control of stress.
- Those who were able to combine teleworking with attending their workplace showed the lowest levels of stress.
